# Microbial Composition and Variability of Natural Marine Planktonic and Biofouling Communities From the Bay of Bengal

**DOI:** 10.3389/fmicb.2019.02738

**Published:** 2019-12-06

**Authors:** Angelina G. Angelova, Gregory A. Ellis, Hemantha W. Wijesekera, Gary J. Vora

**Affiliations:** ^1^American Society for Engineering Education, Postdoctoral Fellowship Program, U.S. Naval Research Laboratory, Washington, DC, United States; ^2^Center for Bio/Molecular Science and Engineering, U.S. Naval Research Laboratory, Washington, DC, United States; ^3^Stennis Space Center, U.S. Naval Research Laboratory, Stennis, MS, United States

**Keywords:** marine biofilm, microbiome, microfouling, planktonic, rRNA

## Abstract

The Bay of Bengal (BoB) is the largest bay in the world and presents a unique marine environment that is subjected to severe weather, a distinct hydrographic regime and a large anthropogenic footprint. Despite these features and the BoB’s overall economic significance, this ecosystem and its microbiome remain among the most underexplored in the world. In this study, amplicon-based microbial profiling was used to assess the bacterial, archaeal, and micro-eukaryotic content of unperturbed planktonic and biofilm/biofouling communities within the BoB. Planktonic microbial communities were collected during the Southwest monsoon season from surface (2 m), subsurface (75 m), and deep-sea (1000 m) waters from six south-central BoB locations and were compared to concomitant mature biofouling communities from photic-zone subsurface moorings (∼75 m). The results demonstrated vertical stratification of all planktonic communities with geographic variations disappearing in the deep-sea environment. Planktonic microbial diversity was found to be driven by different members of the community, with the most dominant phylotypes driving the diversity of the photic zone and rarer species playing a more influential role within the deep-sea. Geographic variability was not observed in the co-located biofouling microbiomes, but community composition and variability was found to be driven by depth and the presence of macro-fouling and photosynthetic organisms. Overall, these results provide much needed baselines for longitudinal assessments that can be used to monitor the health and evolution of this dynamic and critically important marine environment.

## Introduction

The BoB is a semi-enclosed tropical ocean basin in the north-eastern Indian Ocean. It undergoes the strong influence of many natural and anthropogenic factors (severe weather, earthquakes, freshwater inflows, urban, industrial and agricultural development, etc.) (Shetye et al., 1996; Sarma et al., 2016; Sengupta et al., 2016) and is not driven by the specific influence of any particular industry (e.g., petroleum exploration). Despite its dynamic natural features and economic significance, the BoB ecosystem remains one of the most poorly explored marine environments in the world. Cumulatively, these aspects have made the BoB uniquely qualified for the exploration of typical indigenous planktonic and biofouling microbial communities, representing the post-industrial tropical marine environment.

The BoB is characterized not only by its seasonal monsoon-driven circulation, but also by a distinct surface-bound water stratification (within ∼30 m), produced by a significant influx of fresh and cool waters from rainfalls and multiple major river systems (Shetye et al., 1996; Sardessai et al., 2007; Sengupta et al., 2016). During the southwest monsoon (June–September), mixing of the produced water results in penetration of the contained O_2_ and nutrients deeper into the water column (down to ∼200 m) and promotes primary production (Thangaradjou et al., 2012; Vajravelu et al., 2018). Directly beneath this level, the oxygen minimum zone (OMZ) within the BoB is observed extending down to ∼600 m. Beyond that a gradual but limited replenishment of O_2_ occurs, possibly by inflows from the open Indian Ocean (Sardessai et al., 2007). The differences in these environmental conditions within the water column of the BoB are likely to be reflected in the contained microbial communities in terms of their composition, diversity, and functionality.

Despite multiple investigations into the oceanography, phytoplankton dynamics, stratification, and nutrient cycling of this environment (Shetye et al., 1996; Gauns et al., 2005; Thangaradjou et al., 2012; Chen et al., 2013; Jyothibabu et al., 2015; Sarma et al., 2016; Sengupta et al., 2016), there are notably few molecular studies focusing on the microbial composition of the BoB ecosystem (Rao, 2010; Rajpathak et al., 2018) and none exploring non-coastal environments. Recently, large-scale efforts have made marked contributions toward the microbial characterization of multiple marine environments, ranging from the Pacific Ocean, the gyres, the Atlantic Ocean to the deep-sea of the Arctic Ocean (Fuhrman et al., 1993; Sogin et al., 2006; Galand et al., 2009, 2010; Agogué et al., 2011; Jorgensen et al., 2012; Jing et al., 2013; Sunagawa et al., 2015). Although the Indian Ocean was represented in some of these studies (Sunagawa et al., 2015; Jing et al., 2016), they omitted equitable representation of its northern regions, including the BoB. As such, the primary objectives of this project were to provide the first characterization of the BoB’s non-coastal planktonic microbial communities and address this residual information gap. A secondary objective was to similarly interrogate co-located natural biofilm communities to provide a much needed exploration into the natural compositional variability and environmental response of biofilm communities, inhabiting a typical post-industrial tropical marine environment. Familiarity of such microbial profiles, especially for underexplored marine environments, may help sample, site, and season selection for further explorations, as well as address compositional conundrums observed during targeted studies.

High-throughput sequencing has provided the opportunity to explore many complex microbial communities from multiple environments for their true genetic diversity and potential (e.g., marine microplankton, sediments, environmental biofilms). Still, due to the complexity of these systems, the majority of studies have limited their focus to only specific taxonomic groups (e.g., only bacteria or only diatoms), prioritizing resolution over breadth of exploration. As a result, taxonomically integrative community composition studies for many natural microbial communities are still scarce. To mitigate this, the current study utilized targeted sequencing of the V4–V5 hypervariable regions within the rRNA genes of eukaryotes and bacteria simultaneously, thus producing a more comprehensive assessment of the microbial content of the targeted niches. The results not only revealed the composition, diversity, and variability of BoB planktonic and biofouling communities based on depth and geography but also provided insight into the linkage between macro- and micro-fouling communities, utilizing novel differential abundance and co-occurrence analyses.

## Materials and Methods

### Study Site and Sample Collections

**FIGURE 1:**
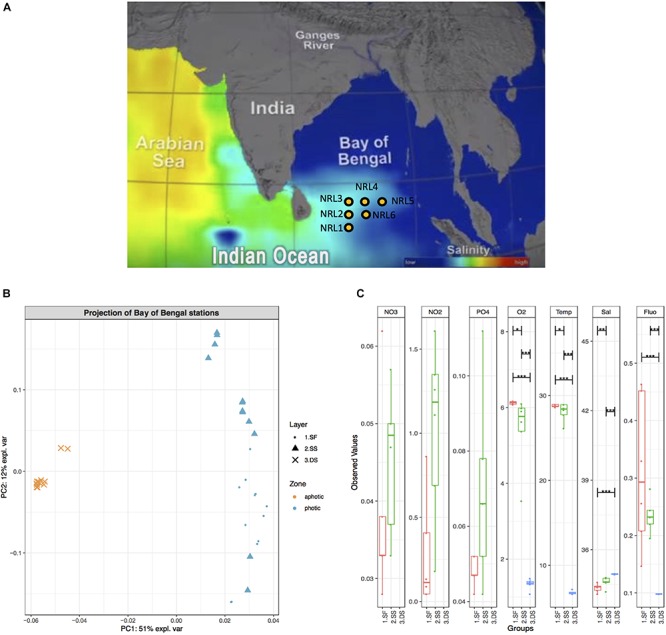
Geographic map of the BoB with hydrographic characteristics. **(A)** Geographic map including salinity gradient. Sampling sites are indicated with yellow dots and labeled NRL1–NRL6. Picture reference: http://podaac.jpl.nasa.gov/SeaSurfaceSalinity/Aquarius. **(B)** PCA ordination plot based on BoB physicochemical characteristics (including oxygen, fluorescence, salinity, temperature, nitrate, nitrite, and orthophosphate content) in August 2015. PC1 explains 51% of variations in physicochemical characteristics and clearly separates deep-sea water (3.DS) from surface and subsurface water (1.SF and 2.SS). **(C)** A boxplot of observed values for physicochemical characteristics of water column in August 2015. Observed values (where available), include temperature [Temp (°C)], salinity [Sal (PSU)], oxygen [O_2_ (mg/L)], fluorescence (Flu), nitrate [NO_3_ (ppm)], nitrite [NO_2_ (ppm)], and orthophosphate [${\text{PO}}_{4}^{3+}$ (ppm)], grouped per examined water layers: surface (red), subsurface (green), and deep-sea (blue). Pairs for comparison of values between layers as well as the significance of this comparison is also indicated (black horizontal bars and stars, respectively). Nitrate, nitrite, and phosphorous content for deep-sea water were not measured.

During the field program for “Effects of Bay of Bengal Freshwater Flux on Indian Ocean Monsoon (EBoB)” conducted by the U.S. Naval Research Laboratory^1^ in 2013, six deep-water moorings were deployed (Figure 1A) to remain suspended within the photic subsurface environment (15–75 m) above the OMZ of the region (∼200 m), for a period of 20 months in the southern BoB (Thangaradjou et al., 2012; Wijesekera et al., 2016b; Vajravelu et al., 2018) at geographic coordinates: 5.009°N, 85.511°E (site named NRL1), 6.500°N, 85.500°E (NRL2), 8.000°N, 85.5°E (NRL3), 7.992°N, 86.990°E (NRL4), 7.992°N, 88.500°E (NRL5), and 6.500°N, 87.000°E (NRL6) (Figure 1A). Each mooring was deployed to maintain an average depth as follows: NRL1 – 44 m, NRL2 – 39 m, NRL3 – 16 m, NRL4 – 45 m, NRL5 – 72 m, NRL6 – 59 m. The ADCP moorings (acoustic Doppler current profiler buoys; Flotation Technologies) were made from Deep-Tech syntactic foam and possessed no antifouling properties. In August 2015, the RV *Roger Revelle* collected the deployed moorings along with water samples and nutrient measurements at depths ∼2 (surface; photic zone), ∼75 (subsurface; photic zone), and ∼1000 m (deep-sea; aphotic zone). Nutrient measurements included Temp, conductivity, pressure, dissolved O_2_, chlorophyll-a (Fluo), and transmissivity (Sal) for the entire water column (at depths 2, 75, and 1000 m) as well as NO_2_, NO_3_, and PO_4_ (${\text{PO}}_{4}^{3-}$) measurements for the layers of the photic zone only (Wijesekera et al., 2016b). Water samples were collected by the ship’s CTD-rosette in duplicates of 1 L volumes and were filtered through GP 0.22 μm Sterivex filters and stored at −80°C. Soft fouling samples scraped from the surface of the moorings and macro-fouling organisms were also collected. Of the scraped samples, six were chosen for microbial community explorations, based on observable differences in fouling material (color, texture, organism association, etc.). Any collected macro-fouling organisms (e.g., barnacles, anemone, mussels, macroalgae, etc.) were excluded from this study.

### Molecular Processing, Library Preparation, and Sequencing

Whole filters or ∼0.03 g of scrapped biofilm material were used for DNA extractions with bead-beating, based on the protocols for DNA isolation accompanying the DNeasy PowerBiofilm Kit (MoBio). PCR reactions were performed based on a two-round PCR process as previously described (Berry et al., 2011). The universal degenerate primers used for this study were chosen as a pair to match the 515F and 927R sequences for 16S rRNA genes of bacterial organisms (e.g., *Escherichia coli*), and 565F and 1150R sequences of 18S rRNA genes from eukaryotic organisms (e.g., *Saccharomyces cerevisiae*) (Hadziavdic et al., 2014; Parada et al., 2016; Caporaso et al., 2018). This primer pair matched 86% of all bacteria, 82% of all archaea, and 70% of all eukarya found in the SILVA SSU 132 reference sequence database (Quast et al., 2013). Fragments amplified with these primers were indexed based on the fusion method scaffolding in a second round of PCR. Indexed libraries were cleaned using Agencourt AMPure XP Beads (Beckman Coulter) and pooled together to be loaded into an Ion 530^TM^ Chip in an Ion Torrent Chef System (ThermoFisher Scientific). Along with all environmental samples, Microbial Community DNA standards (ZymoBioMics) were processed similarly, to assess PCR bias (Supplementary Figure S1). Sequencing was performed bi-directionally with 400 bp chemistry via the Ion Torrent S5 System (Supplementary Figure S7).

### Bioinformatic Analyses

Reads were examined for quality using FastQC and quality screening, trimming, and filtration (pre-processing) were performed using PRINSEQ and TagCleaner software (Andrews, 2010; Schmieder et al., 2010) to quality score >25, mean quality >20, and length >150 bp (Supplementary Table S1). Read orientation was determined using SILVA database (v132) (Quast et al., 2013). Forward (F) and reverse (R) read were separated using USEARCH (v10.0) (Edgar and Flyvbjerg, 2015). Individual sets of representative F and R sequences were prepared, using only the longest reads (>250 bp) and the UNOISE algorithm (Edgar and Flyvbjerg, 2015). These representative sets were then assembled into full amplicons using cap3 software (Huang and Madan, 1999) at 99% similarity threshold and allowance of 90% read overhangs. The produced 5,074 contigs were used as the representative operational taxonomic units (OTUs) for the study, to which the prepossessed reads for each sample were mapped (Supplementary Table S1). Taxonomic assignments of the produced representative OTUs were performed using the SINTAX algorithm (Edgar, 2016) and SINTAX-reformatted database, containing a manually curated combination of the following published databases: SILVA (version 132) (Quast et al., 2013), Ribosomal Database Project (RDP version 11) (Cole et al., 2014), Protist Ribosomal Reference database (PR^2^ version 4.1) (Guillou et al., 2013), and the Plastidal 16S rRNA Gene Database of Photosynthetic Eukaryotes (PhytoRef version 1.0) (Decelle et al., 2015). Relevant OTUs with unassigned taxonomy were also manually subjected to BLAST (BLAST + v2.8.0) against NCBI’s nr and 16S Microbial database (2 May 2018 release) (Camacho et al., 2009; Supplementary Figure S7). OTUs with taxonomic identification are herein also referred to as “phylotypes.” Sequences have been deposited to NCBI’s Sequence Read Archive (SRA) and are available under BioProject number PRJNA498301.

### Statistical Analyses

Downstream filtering and biostatistical analyses were performed in R (R Development Core Team, 2010) via R Studio (RStudio Team, 2015). Supporting packages and software used were as follows: for sample filtering and microbial profiling visualization – “phyloseq” and Krona tools (Ondov et al., 2011); general sample biostatistics, community structure, and bio-bio analyses – “vegan” and “sinkr” (Clarke and Ainsworth, 1993; Oksanen et al., 2018); differential analysis between samples: “MixOmics” and “metagenomeSeq” (González et al., 2012; Paulson et al., 2013; Rohart et al., 2017). Significance threshold for all tests was selected as 0.05 (*p*-values). OTUs with low abundance (<10) and low presence across samples (<30%) were excluded from the study. Samples with <20,000 reads/individuals were also excluded from the study. Planktonic samples were analyzed on depth of sampling (photic zone surface, photic zone subsurface, or aphotic zone deep-sea for planktonic communities) and geographic location (NRL 1–6). Biofilm samples were examined based on geographic location, depth, and nutrient features of each site. Further comparative analyses between biofilms were performed based on a subset of biofilms (*n* = 12), selected for distinguishable clustering during non-metric multidimensional scaling (nMDS).

## Results

### Physiochemical Properties of the BoB Water Column

The most significant differences in physicochemical characteristics of the BoB during August 2015 were between the photic (surface and subsurface) and aphotic (dep-sea) zones (Figures 1B,C) with one principal component (PC1) explaining more than half (51%) of the variations (Figure 1B) and clearly separating the two groups (global PERMANOVA *F*-model = 3583 and *p*-value = 0.001). A significant difference between the surface and subsurface layers was also observed (PERMANOVA *p*-value = 0.0003), however, at a lower mean variance and level of separation than the deep-sea contrasts (*F*-statistic = 7.6 for surface vs. subsurface waters, *F*-statistic = 17,358 and 2,393 for deep-sea to surface and subsurface, respectively). While depth comparisons revealed significant differences between water layers, the physicochemical properties of the sites throughout the entire water column showed little differentiation (global PERMANOVA *p*-value = 0.99; Supplementary Figure S2A). Even comparisons of the sites only within the photic zone showed no significant nutrient variation (global PERMANOVA *p*-value = 0.16; Supplementary Figure S2B).

### BoB Planktonic Microbial Communities

**FIGURE 2:**
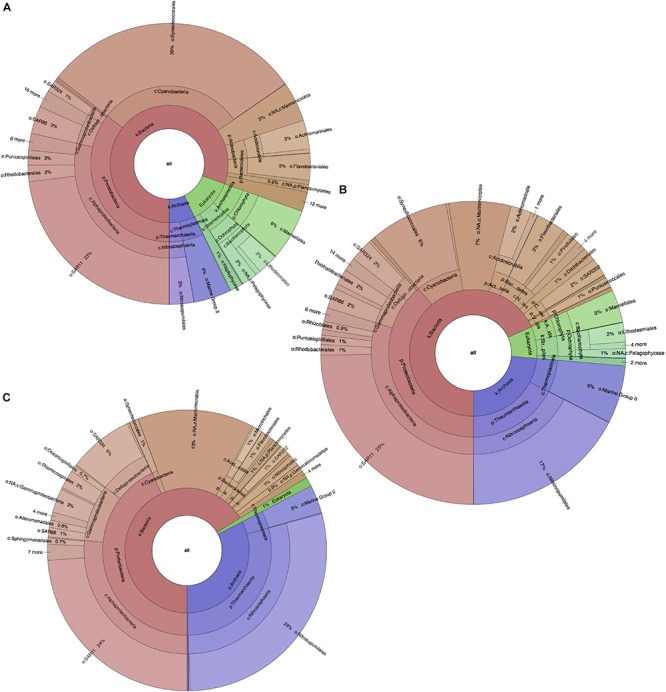
Planktonic microbial profiles for each BoB water layer, based on 16S and 18S rRNA gene amplicons and visualized with Krona Tools. **(A)** Photic zone, surface layer, 1.SS at ∼2 m depth. **(B)** Photic zone subsurface layer, 2.SS, at ∼75 m depth. **(C)** Aphotic zone, deep-sea layer, 3.DS at ∼1000 m depth. Taxonomic groups were assigned and represented to deepest taxonomic level possible.

Similarly to the physicochemical differences within the water column, significant differences in taxonomic composition were also found between all water layers (PERMANOVA *p*-value = 0.0001), along with a similar distinguishability pattern (Supplementary Figures S3C,D). These were contributed primarily by variations in algal and archaeal species such as *Synechococcus*, *Prochlorococcus*, *Ostreococcus*, and *Nitrosopumilus* (cumulatively 45–49% of all community differences between all layers; SIMPER analysis). These contributions, however, showed opposite trends in terms of variation, as photosynthetic organisms (*Synechococcus*, *Prochlorococcus*, *Ostreococcus*) decreased in abundance with depth (from ∼42%, to ∼17%, to ∼2% for each level) probably due to limitations in light penetration, while reads from archaeal organisms (*Nitrosopumilus*) drastically increased with depth (from ∼7 to ∼24 to ∼33%; Figure 2). From the bacterial domain, the most reads were found to be from Proteobacteria, Cyanobacteria, Marinimicrobia (SAR406), Bacteroidetes and Actinobacteria (Actinomarina). While Cyanobacteria (∼30%) quickly decreased with depth, Marinimicrobia increased from 5 at the surface to 7 to ∼13% at deep-sea level. Proteobacteria were also found to increase with depth (36–39–43%) with this change being primarily due to the increased fraction of Gammaproteobacteria and Deltaproteobacteria (5–6–9% and 2–3–8%), rather than a decrease in Alphaproteobacteria. Alphaproteobacteria maintained a stable dominance within the entire microbial community at any depth (27–30%), with SAR11 (*Pelagibacter ubique*) having greatest presence and occupying a stable quarter fraction. Changes were also observed in the Bacteroidetes phylum (primarily Flavobacteria), which decreased from 4% in the photic zone to ∼2% in the aphotic zone.

Despite little physicochemical difference between sites, geographic variation in the planktonic microbial communities was strongly observed. Geographic variations were found to have a significant effect within both layers of the photic zone, contributing as much as 78% of the observed variation between planktonic communities (PERMANOVA *p*-values = 0.001, ANOSIM *R*-values = 0.76 and 0.78, *p*-values = 0.001). The community variation between locations in the photic zone was also evident within the taxonomic profiles, where variation in most abundant phylotypes was observed from site to site (Supplementary Figures S4A,B). In contrast to the photic layers, the deep-sea layer (3.DS; aphotic zone) did not present significant levels of geographic variation (PERMANOVA *p*-value = 0.3, ANOSIM *R*-value 0.32, *p*-value = 0.03). Again, this could also be seen in the site-based microbial profiles (Supplementary Figure S4), where community composition of the aphotic zone appeared stable across sampling sites (Supplementary Figure S4C). Another interesting observation from the exploration of the community profiles was revealed by the PCoA plots of weighted vs. unweighted UniFrac distances from the depth-differentiated communities. The weighted plot (Supplementary Figure S3B) produced more dispersed photic layer community samples, than those of the unweighted plot (Supplementary Figure S3A), indicating that community variation within the photic zone was driven by the most abundant phylotypes. Meanwhile, within the aphotic zone, it was the rarer members that accounted for a more influential role.

Another noteworthy observation from the site-based community exploration was the increased abundance for variants of the species *Prochlorococcus marinus* found at location NRL5 (Supplementary Figure S4). This variation was observed in all replicates for both layers within the photic zone, suggesting this phenomenon was an actual *P. marinus* bloom, rather than an artifact of PCR amplification (PCR bias). While the observed abundance peak could not be associated with a notable increase in any of the measured nutrient concentrations at that site (Supplementary Figure S2), it is possible it could be attributed to differences in non-measured factors (e.g., other nutrients, dissolved organic matter, pH, etc.).

**FIGURE 3:**
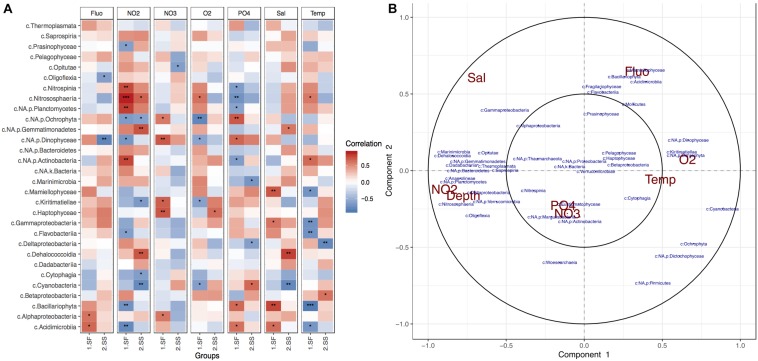
Physical and chemical factors influencing BoB photic planktonic microbial communities. **(A)** Pearson correlation heatmap for the top 30 most abundant taxonomic classes and environmental variables per layer. Stars represent significance of correlation. **(B)** Circle correlation plot, where relationships between phylotypes (blue) and environmental factors (red) are presented in terms of radial distances and their cosine angles (González et al., 2012; Rohart et al., 2017). Sharp and obtuse angles between variables represent positive and negative correlations, respectively, while perpendicularity presents no correlation. Strength of correlations is represented by distance from the radial origin.

Correlation analyses performed between the most abundant taxonomic classes and measured environmental variables indicated that NO_2_ was the most influential chemical factor on planktonic microbial composition in the surface, while O_2_ levels strongly affected the subsurface communities (Figure 3). The strongest dependency to the former was exhibited by members of Nitrospina, Planctomycetes, Actinobacteria, and Nitrososphaeria classes (Figure 3A), along with Dadabacteria, Bacteroidetes, and Proteobacteria (Figure 3B). Algae-like diatoms showed significant negative correlation to the presence of NO_2_. The increase of Fluo within the photic zone of the BoB was related to the presence of algae, but the correlation analysis revealed specific taxonomic groups such as Mammiellophyceae, Bacillariophyta, Prasinophytes, and Haptophytes. O_2_ levels, which decreased with depth, showed most significant effect in the deep-sea layer and negative correlation with some chemolithotrophic phylotypes such as Nitrososphaeria, Marinimicrobia, some Gammaproteobacteria (especially orders Chromatiales, Thiotrichales), and Deltaproteobacteria (SAR324; Supplementary Figure S2C). An increase in Temp was positively correlated with most taxonomic groups within the deep sea. NO_3_ and phosphate were shown to have the strongest influence on photosynthetic organisms such as Dinophyceae (Dinoflagellates), Bacillariophyta, Ochrophyta, Cyanobacteria, Eustigrmatophyceae, and Pelagophyceae, but the direction of influence depended on the layer (Figure 3).

### BoB Biofouling Microbial Communities

**FIGURE 4:**
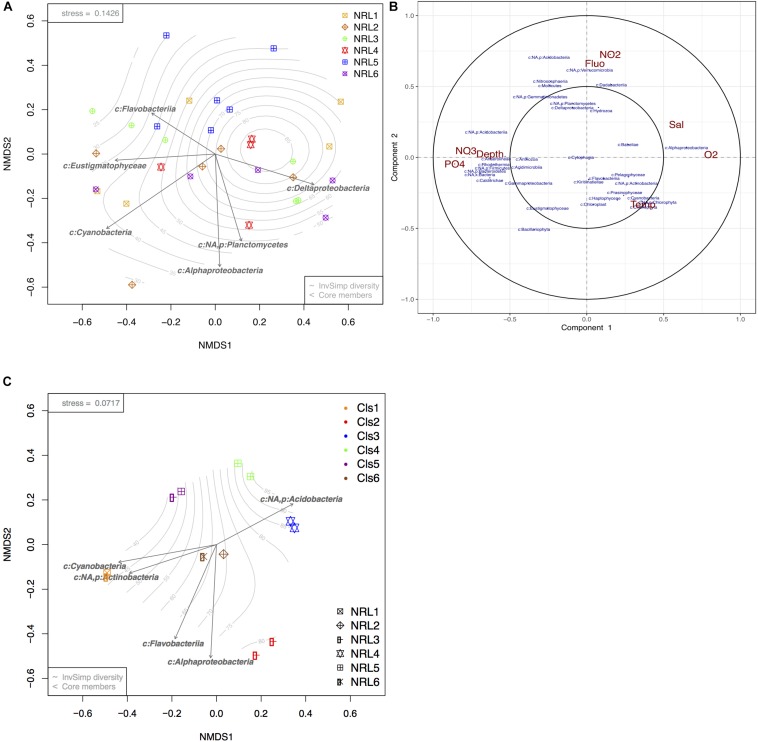
Beta diversity analyses of BoB biofilm communities and microbial response to environmental variables. **(A)** nMDS ordination plot of square root transformed Bray–Curtis dissimilarity matrix produced from photic zone subsurface biofilm communities (*n* = 31). **(B)** Circular correlation plot of BoB biofilm microbial community members (blue) at each site and depth and corresponding environmental variables (red) presented in terms of radial distances and their cosine angles (González et al., 2012; Rohart et al., 2017). **(C)** nMDS plot of square root transformed Bray–Curtis dissimilarity matrix produced from 12 selected biofilms. Arrows present core phylotypes (significance > 0.05) that best explain the biotic structure with length representing the strength of the correlation. Gradient represents sample diversity based on Inverse Simpson values.

Biofouling microbial communities collected from the moorings at the six BoB sites were drastically more variable from each other, with species richness and diversity being higher than those of planktonic communities (Supplementary Figures S5A,B). Additionally, communities that belonged to the same site (and mooring) could present with contrasting community composition, whereas communities belonging to different sites could present strongly similar compositions (Figure 4A). Despite the high variation between communities, a bio-bio community analysis (based on Mantel tests; Clarke and Ainsworth, 1993) identified certain phylotypes that could best explain the biotic structure of the diverse biofouling communities. The most significant members of these were found to be Eustigmatophyceae (micro-algae; Stramenopiles), Cyanobacteria, Flavobacteria, Delta proteobacteria, Alphaproteobacteria, and Planctomycetes (Figure 4A). Initially, a significant factor driving these differences was geography (PERMANOVA *p*-value = 0.001, ANOSIM *R*-value = 0.22, *p*-value = 0.004). However, pairwise comparisons between sites (pairwise PERMANOVA) showed only 4 out of 15 site vs. site comparisons to have significant results, all of which included site NRL5 (the most east-bound site, Figure 1A).

Apart from the geographic variation, community correlation analyses showed that the depth of moorings and nutrients (Figure 4B) had a strong influence on biofilm community composition, but no effect on richness or diversity (ANOVA *p*-values >> 0.05). Increasing depth corresponding with NO_3_ and phosphate enrichment showed a significant positive correlation to taxonomic groups such as Bacteroidetes, Acidobacteria, Acidimicrobiia, and Gammaproteobacteria, while negatively impacting Alphaproteobacteria and Dadabacteria. Alphaproteobacteria showed high positive correlation with increased O_2_, Temp, and depth. Temp showed the least variation between the moorings, but demonstrated a positive influence on most photosynthetic organisms integrated within the biofilms (Haptophyta, Prasinophyta, Chlorophyta, Bacillariophyta, Cyanobacteria; Figure 4B). Planctomycetes, Deltaproteobacteria, Verrucomicrobia, and Dadabacteria showed the strongest positive correlation with NO_2_ content and strong negative correlation with planktonic algae (Fluo).

**FIGURE 5:**
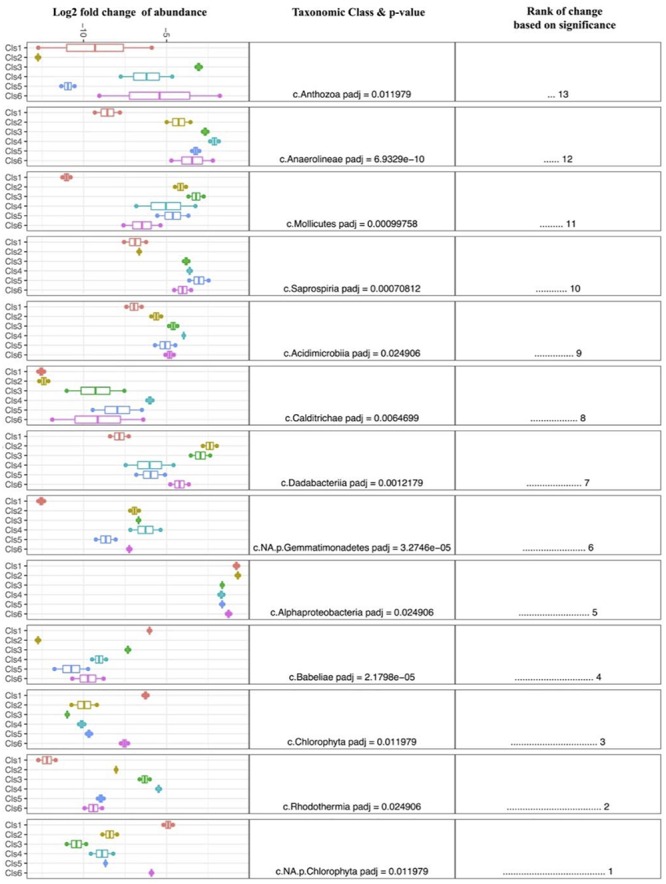
Differential abundance analysis (DESeq2) between clusters of biofilm samples, exploring and ranking the taxonomic classes that were most significantly different between clusters. A larger fold change is indicative of enrichment within each group.

**FIGURE 6:**
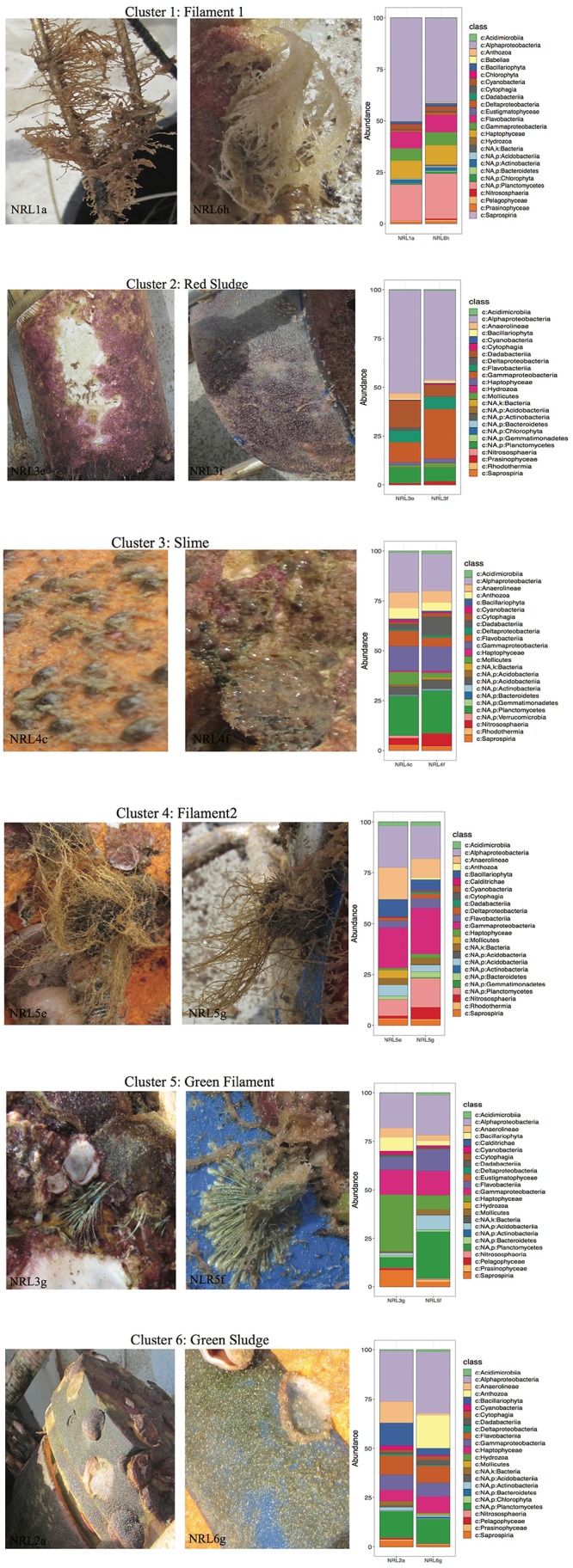
Phenotypes of selected samples along with the profiles of their top 25 most abundant species from the collected biofilms. Macrofouling organisms observed were not examined in this study. Taxonomy was presented to the deepest assigned rank.

Despite the high variability between biofouling communities, 12 samples were chosen for further examination, based on the noted closeness within the nMDS ordination plot (Figure 4C). The 12 samples were partitioned into six Cls with microbial communities between Cls showing significant differences (Figure 4B, *p*-value = 0.001), with Cls differentiation explaining as much as 90% of the community differences (ANOSIM *R*-value = 0.89, *p*-value = 0.001). Core community members explaining the biotic structure of the communities from each Cls (Figure 4C) included Acidobacteria (Cls 3), Cyanobacteria and Actinobacteria (Cls 1), Flavobacteria (Cls 6), and Alphaproteobacteria (Cls 2). Differential abundance analysis between Cls revealed Cls-distinguishing phylotypes (Figure 5), and revealed Rhodothermia and Anaerolineae as significantly enriched in Cls 4 and Saprospiria in Cls 5. Dadabacteria was noted as significantly enriched in Cls 2. Cls 1, 2, and 6 were richest in colonizing Proteobacteria (Figure 6 and Supplementary Figure S6). Cls 2 and 6 showed an enrichment from the Rhodobacterial *Ruegeria* species, while Cls 1 had generally lower Alphaproteobacterial diversity and was enriched in *Amylibacter* species (Figure 6 and Supplementary Figure S6). Cls 3 communities were noted for being highest in community evenness (evenness index = 0.78). Cls 4 was highest in diversity and presented an enrichment of Gammaproteobacteria (especially order Arenicellales). The communities collected from Cls 5 were rich in *Phaeocystis antarctica* (Haptophyceae), *Ditylum brightwellii* (Bacillariophyta), Pelagophytes, and Eustigmatophytes (Figure 6 and Supplementary Figure S6).

The examination of samples within each Cls showed that the source material of the Cls often had similarities in their macroscopic features (Figure 6). This was especially true for samples within Cls 3 (only 15% difference in community composition between samples), that were collected from the same mooring area, noted as rich in macro-fouling organisms (i.e., Cnidarians). Another Cls with high similarity of its samples was Cls 1 (only 19% different; SIMPER), which came from the surface of macrofouling algae (filamentous material), despite differences in site (NRL1 and NRL6) and depth (44 and 59 m, respectively). Samples forming Cls 4 were also observed to come from macro-algae with very similar features as those of Cls 1 (Figure 6), however, were demonstrated to be more different from each other (28%; SIMPER), despite being collected from the same mooring. Additionally the two Cls showed 54% difference between each other (SIMPER), despite an obvious high similarity in source material, demonstrating that material features did not always correspond to community resemblance. Samples from Cls 2 and 6 (red and green sludge), collected directly from a mooring area of sparse macrofouling (abiotic surface), showed relatively low discrepancy between in-group samples (22 and 38%, respectively) and ∼52% discrepancy between the Cls. Samples from Cls 6 were noted to belong to different moorings with high depth discrepancy (20 m). The highest differences within a Cls (64%) were found between the communities collected from green filament (presumably hydroid or another algae; Cls 5), which were also collected from different moorings at the most contrasting depths (NRL3 at 16 m and NRL5 at 72 m) and showed higher community discrepancy (64%).

## Discussion

The microbial profiling conducted in this study was produced using “universal” PCR primers, selected for their ability to simultaneously target both 16S and 18S rRNA genes. In an effort to achieve broad-range profiling, the selected pair of primers were not optimized for detailed representation of all eukaryotic organisms (Caporaso et al., 2011; Hadziavdic et al., 2014), and were even found to be relatively insensitive to the presence of DNA from some crucial marine planktonic and biofilm constituents such as Bacillariophyta (diatoms; see the section “Materials and Methods” and Supplementary Figure S1). Amplification-based molecular methods are often more problematic for eukaryotic organisms, due to the increased variability of the targeted 18S rRNA gene (Salta et al., 2013). As an attempt to compensate for this deficiency, this study also referenced a plastidal database for taxonomic assignments of photosynthetic eukaryotic organisms, allowing improved resolution for algal characterization, based on chloroplast 16S rRNA genes. Moreover, detailed examinations of the BoB phytoplanktonic content, variability, and influencing factors are being regularly performed via other means (Jyothibabu et al., 2015; Sarma et al., 2016; Vajravelu et al., 2018). Another constraint within the study arose from its objective to explore the microbial biosphere within the BoB. Although macro-fouling organisms are a major component of both planktonic and biofouling environments (e.g., Arthropods, Cnidarians, macro-algae) (Zaiko et al., 2016; Debroas et al., 2017; Briand et al., 2018), the techniques used here were not optimized for their exploration, but rather focused on the microorganisms coexistent within their habitats. Therefore, the taxonomic inclusivity of the primers was used to obtain the composition, variability, and environmental response of BoB microbial planktonic communities, as well as microbial fouling communities, formed on submerged surfaces with no artificial antifouling properties.

### BoB Water Column and Planktonic Communities

As the samples in this study were collected during a research expedition to obtain high-resolution hydrographic field maps of water exchange in the region between the BoB and the Arabian Sea (south-central BoB), the sampling sites were chosen to target a specific region of high hydrodynamic activity (Wijesekera et al., 2016a, b). Despite observed physicochemical similarity between all sites, the microbial communities of site NRL5 (both planktonic and biofilm) were found to carry characteristic signatures, not observed at the other five sites (i.e., cyanobacterial blooms for the planktonic community and site-based distinguishability for the biofilms). As NRL5 was at the furthermost border of the targeted exchange region, it remains possible that the communities in this site were responding to unmeasured factors (e.g., seasonality, depth). Within the planktonic community, a seasonal response could be a reason for these differences, as the pre-monsoon season in the BoB has been noted for bringing about annual phytoplankton maxima, due to increased inflow of nutrients and freshwater (Thangaradjou et al., 2012; Vajravelu et al., 2018). In addition, and partly due to seasonality, is also the influence of colder, nutrient-rich water transported from the deeper layers to the euphotic zone by the strong and prolonged presence of seasonal eddies (Chen et al., 2013; Jyothibabu et al., 2015; Wijesekera et al., 2016b).

Recently, Rajpathak et al. (2018) reported on amplicon-based analyses of BoB coastal planktonic microbial communities inherent to OMZ and non-OMZ. Consistencies in the findings of the two studies were noted within the taxonomic profiles of the deep-sea BoB layers and included the high abundance of organisms such as *Nitrosopumilus* and SAR11 suggesting similar deep-sea communities between coastal and open-ocean locations. Although measurements for some crucial nutrients were not taken for this study at the deep-sea level (NO_3_, NO_2_, and organic phosphate), other BoB oceanographic studies report of their increased levels at ∼1000 m (Sverdrup et al., 1942; Sardessai et al., 2007; Bristow et al., 2017). This supported the observed abundant presence of the chemolithotrophic organisms (Archaea, Marinimicrobia, SAR324, Gammaproteobacteria) within this O_2_ and light deprived environment (Orcutt et al., 2011; Wright et al., 2012). Many other previous deep-ocean community observations report of high similarities between deep-ocean communities, not only within the east Indian Ocean, but also at larger distances expanding even to different oceanic regions (Jing et al., 2013, 2016). The results of the current study, in line with previous reports, suggest that in the deep-sea, biogeography is drastically less pronounced than at the surface. Surface-based profiles, however, contrasted with those of the coastal studies of Rajpathak et al. (2018). In the coastal photic subsurface layers of west BoB, high abundance of gammaproteobacterial Alteromonadales and Oceanospirilalles variants (*Alteromonas*, Halomonas, *Pseudoalteromonas*, and *Pseudomonas*) were reported. These organisms are recognized for their copiotrophic and hydrocarbonoclastic properties (Paissé et al., 2008; Niepceron et al., 2013; Chronopoulou et al., 2015) and are indicative of a pronounced anthropogenic impact on the coastal BoB environment (Elifantz et al., 2013; Celikkol-Aydin et al., 2016). In contrast, the photic layers of the south-central BoB explored in the current study demonstrated undetectable levels of the listed gammaproteobacterial variants, yet a high abundance of SAR11-associated species. Since the latter are ubiquitous within the oligotrophic marine environment, and sensitive to increased carbon and nutrient loads (Jing et al., 2013; Giovannoni, 2017), their stable presence within the BoB water column was suggestive of a photic zone environment unperturbed by the same anthropogenic factors as the coastal regions. Further in contrast to coastal surface and eutrophic (nutrient enriched) regions, Gammaproteobacteria in the photic zone of the BoB were found to have a different internal composition, not dominated by Alteromonadales and Oceanospirillales (Dang and Lovell, 2002; Gregoracci et al., 2012; Elifantz et al., 2013; Celikkol-Aydin et al., 2016; Jing et al., 2016), but by Thiohalobacteria and the SAR86 clade. These groups are known chemolithotrophic bacteria (utilizing sulfur and various metals) typically thriving in oligotrophic marine environments (Sorokin et al., 2010; Dupont et al., 2012). Conversely, community composition very similar to those found in the current study [i.e., with surface waters being predominated by Proteobacteria (especially Alphaproteobacteria) and Gammaproteobacteria increasing with depth] have been reported within various epipelagic environments (Agogué et al., 2011; Friedline et al., 2012; Gregoracci et al., 2012; Sunagawa et al., 2015; Jing et al., 2016).

Results from the planktonic community analyses in this study also indicated that nutrient availability (primarily NO_2_ and phosphate) were the most dominant factors in vertical community variations, with the surface community (as most oligotrophic) being most strongly affected. This result complements the findings of multiple other studies in the BoB that report that nutrients have significant impact on phytoplankton and bacterioplankton (Thangaradjou et al., 2012; Chen et al., 2013; Sarma et al., 2016; Vajravelu et al., 2018). Nutrients as a driving factor were also reported in the surface waters of the second largest tropical bay in the world – Guanabara Bay (Gregoracci et al., 2012), but not in temperate-climate regions such as the Chesapeake Bay (Kan et al., 2006). In addition, observations on global epipelagic microbiomes also report Temp as the driving factor of vertical microbial variations (Sunagawa et al., 2015). These reports suggest that at least within tropical bay environments, it is nutrient availability, not Temp, that drives microbial composition. This observation can be explained by the relatively higher and more annually stable Temp within tropical regions (compared to temperate ones), which affect the flux rates of nutrients into the water (Asmus et al., 2000; Giovannoni and Vergin, 2012).

### Biofouling Communities

The results from biofouling community comparisons suggested that site specificity was not a major driving factor for community variability. Despite global PERMANOVA tests showing high significance between samples in site-based categories, pair-wise site comparisons of communities showed little significance between samples from different sites, suggesting that geographical distinction cannot be reliably concluded. This was in sharp contrast with the planktonic communities, which showed clear geographic variation within the photic zone. The exception was site NRL5, where statistical tests indicated strong location-based distinction (Figure 4A and Supplementary Figure S5B). As part of a high-resolution hydrographic monitoring effort for the region, however, mooring sites were intentionally chosen to have relatively similar physiochemical and hydrodynamic properties, but allowed moorings to suspend in different and variable depths between sites (Wijesekera et al., 2016b). As site and depth variations for the moorings were correlated (each mooring stood at a different site and depth), depth was suspected to play a larger role in biofilm community variability, than geography. The mooring at site NRL5 was suspended at a depth greater than any other mooring from the explored sites (∼72 m on average) (Wijesekera et al., 2016b). Correlation analyses showed that depth had a substantial influence on the community composition, as it was linked to increase in nutrient levels which asserted that it was likely a more influential factor than geography. Biofilm diversity and richness, however, were not found to change significantly between the explored depths, which is contradictory to previous findings (Bellou et al., 2012). However, a simple explanation could be that the explored depths between moorings in this study did not vary from each other significantly enough to affect community diversity and richness.

As expected from previous studies, the co-located biofouling communities proved to be greatly different biotic assemblages than their planktonic counterparts with increased variability (looser clustering) even within a site (Jones et al., 2007; Salta et al., 2013; Pepe-Ranney and Hall, 2015; Celikkol-Aydin et al., 2016). Marine biofilms have been reported to be generally predominated by Proteobacteria (primarily from the alpha and gamma groups), Bacteroidetes (especially Flavobacteria), and micro-algae, and the results from the current study did not deviate from these observations (Salta et al., 2013; Celikkol-Aydin et al., 2016; Dang and Lovell, 2016). Proteobacteria found predominating the mooring surfaces included the most common colonizers from the *Roseobacter* clade (Rhizobiales, Rhodobacterales, Rhodospirillales) known for their presence and activity in warm marine coastal environments, polymer degradation, biofilm formation, and sulfur oxidation. These Alphaproteobacteria have also been recognized for algal association, anoxygenic phototrophy, and functional efficiency despite low nutrient availability (Buchan et al., 2005; Bruhn et al., 2007; Brinkhoff et al., 2008; Elifantz et al., 2013; Salta et al., 2013), which explains their seemingly negative correlation with nutrients like NO_3_ and phosphorus (Figure 4B). Gammaproteobacteria from the order Alteromonadales are also very highly associated with efficient surface colonization (Dang et al., 2008; Salta et al., 2013), but such phylotypes were not identified as abundant community members in this study. This may have been due to limitations in the taxonomic assignment rather than their actual absence, as many Gammaproteobacterial variants remained unassigned. Instead communities were found abundant in Gammaproteobacterial species from the order Chromatiales. Also known as purple-sulfur bacteria, Chromatiales species are anoxygenic photolithoautotrophic and photoorganoheterotrophic organisms capable of nitrogen fixation and oxidation of hydrogen sulfide under anaerobic conditions (Proctor, 1997; Imhoff, 2005; Salta et al., 2013; Behera et al., 2014). They tend to be inhibited by O_2_ and rely greatly on nutrient availability, which would explain their observed relationship with O_2_ and nutrients within the BoB biofouling communities. Flavobacteria (more specifically order Flavobacteriales) and Planctomycetes (order Planctomycetales), commonly found within marine biofilms, are known for their association with algae, due to their preference for algal polymers as an energy source (Fernández-Gómez et al., 2013; Lage and Bondoso, 2014). Concurrently, Flavobacteria in particular showed a very high association with the algae within the BoB biofouling communities. Algae, especially diatoms and Cyanobacteria, are also common contributors to biofouling communities. Algae are efficient producers of extracellular polymers and long-chain fatty acids (of interest to Flavobacteria and Planctomycetes) and often have a palmelloid life stage compelling them to aggregate on submerged surfaces (Sanmartin et al., 2010; Rivas and Riquelme, 2012; Salta et al., 2013; Pepe-Ranney and Hall, 2015; Celikkol-Aydin et al., 2016). Interestingly, Eustigmatophyceae (e.g., *Nannochloropsis*) have appeared as core members of BoB biofouling communities explored in this study. These organisms are found to be primarily planktonic in the marine environment, with no previous evidence for their presence in biofilms, however some members are found associated with macro-algae. Their ecological role within the biofouling communities might also be associated with their efficient production of high-molecular weight lipids [fatty acids, hydrocarbon polymers, and carotenoids (of interest to Bacteroidetes)], and high association with the *Roseobacter* clade (Buchan et al., 2005; Not et al., 2012; Eliá et al., 2017). *Acidobacteria*, found in the Cls 3 biofilm communities, are known for their association with Cnidarian organisms and efficient use of NO_2_ as a nitrogen source (O’Connor-Sánchez et al., 2014; Kielak et al., 2016). In addition, these organisms are also known as efficient extracellular polymeric substance producers and common members within biofilms (O’Connor-Sánchez et al., 2014). Lastly, Deltaproteobacteria, also found as core members of BoB biofilms, are known sulfate-reducers in O_2_-poor marine environments and commonly associated with sedimentary particles and biofilms (Miyatake et al., 2009; Jones et al., 2010; Salta et al., 2013; Auguet et al., 2015). Overall, the findings from the known metabolic capacities of core community members indicate that processes within the BoB biofilms were related to photosynthesis or sulfur and nitrogen utilization, depending on nutritional, O_2_, and light availability.

An interesting observation from this study was the relationship between community structure and material source. The most closely related communities tended to be epibiotic (growing on the surface of a macro-organism). As epibiotic biofilms often act as a “second skin” to the macro-organism they surround, their composition is often influenced by the host (Wahl et al., 2012). Not surprisingly, this means that these communities would be less dependent on environmental factors such as depth and light availability, as indicated by the observed differences between samples within Cls 1 and 5. As for the biofilms on the abiotic surface, these were found to carry more similarities when collected from similar environmental conditions (e.g., Cls 2) and a bit more variable when differences in depth and nutrients were observed (e.g., Cls 6).

## Conclusion

The findings from this study provide the first insights into the properties and composition of BoB planktonic and microbial biofouling communities, along with their response and adaptations to the environment. As our findings demonstrate that these communities are shaped by complex interactions among species and environmental factors and as such, provide needed baselines for longitudinal assessments that can now be used to monitor the health and evolution of the BoB. A functional metagenomic analysis of these same planktonic and biofouling communities is now underway to provide additional insight into their taxonomic structure, metabolic potential, and ecological role.

## Data Availability Statement

The datasets generated for this study can be found in the sequences that have been deposited to the NCBI Sequence Read Archive (SRA) and are available under BioProject number PRJNA498301.

## Author Contributions

AA, HW, and GV designed this study. GE collected the samples. AA performed the experiments and bioinformatic analyses, and wrote the manuscript. GV provided the support and supervision during manuscript drafting. GE, HW, and GV contributed to the writing by providing suggestions and helping with the revisions. HW was the research vessel science director. All authors reviewed and approved the final version of the manuscript.

## Conflict of Interest

The authors declare that the research was conducted in the absence of any commercial or financial relationships that could be construed as a potential conflict of interest.

## Footnotes

1 Part of an international effort by the Air-Sea Interactions in Northern Indian Ocean (ASIRI) of the U.S. Office of Naval Research, the Ocean Mixing and Monsoons (OMM) of the Monsoon Mission of India, and the Coastal Current Observations Program (CCOP) of Sri Lanka (Lucas et al., 2014; Wijesekera et al., 2016a, b).

2 In the current study, the chosen primers showed 44% coverage of fungal organisms and ∼2% coverage of Ochrophyta organisms, as determined by SILVA TestPrime v1.0 analysis against SILVA SSU v132 database. Similar findings were reported by Parada et al. (2016).

## Abbreviations

BoB: Bay of Bengal
Cls: cluster
Fluo: fluorescence
NO_2_: nitrite
NO_3_: nitrate
O_2_: oxygen
PO_4_: orthophosphate
Sal: salinity
Temp: temperature.
