# Supplementary material for: Microbial Composition and Variability of Natural Marine Planktonic and Biofouling Communities From the Bay of Bengal
Source: Front Microbiol. 2019 Dec 6;10:2738. doi: 10.3389/fmicb.2019.02738 (PMC6908470; doi:10.3389/fmicb.2019.02738)
Supplement: Supplementary file 1 [file Data_Sheet_1.pdf]

Supplemental Graphs and results:

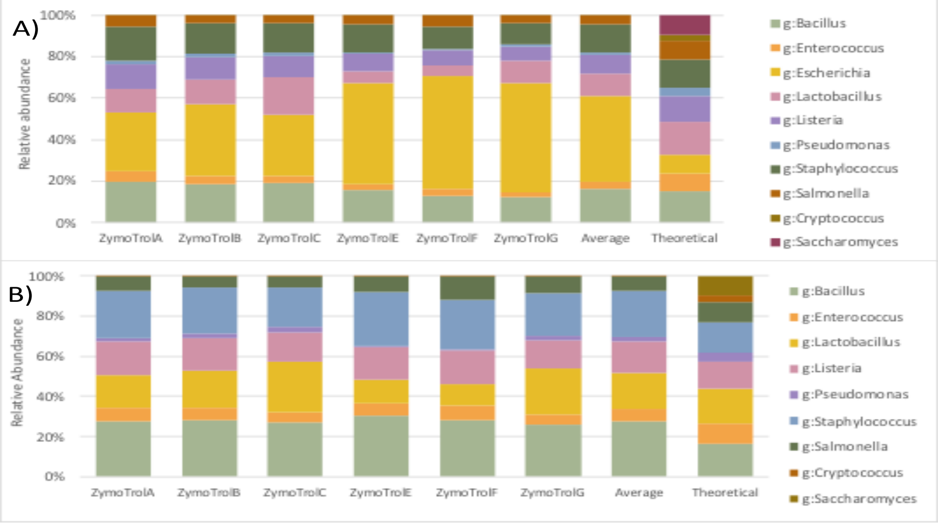

Figure S1. Community profiles of mock communities (ZymoBioMics controls) produced via 16S rRNA amplicon sequencing of the V4-V5 hypervariable region and taxonomic assignment down to genus level. A) Community profiles of complete communities. B) Community profiles of same communities with E. coli associated variants removed from analysis for visualization and bias assessment purposes. Profiles include 6 control samples, their averaged profile (average) and a theoretical (expected) profile. Samples include 2 biological replications (samples ZymoTrolA and ZymoTrolE), and 2 technical replicates for each biological replicate (Profiles for ZymoTrol B and C are technical replicates of ZymoTrolA and Profiles for ZymoTrol F and G are technical replicates of ZymoTrol E). Results showed bias specifically towards E. coli associated variants. Similar bias, was not observed towards the closely related organisms such as Salmonella enterica (Figure S1A). As E. coli-associated variants were not found excessively in any sample (<1% occurrence), this bias was not considered consequential to the study. Likely due to primer sensitivity [1], control communities also showed the underrepresentation of some eukaryotic organisms such as diatoms (Stramenopiles) and fungi (Opisthokonta). The presence of chloroplast 16S rRNA gene in the chloroplasts of diatoms, along with the utilization of the PhytoRef database for taxonomic assignments was used to partially compensate for this deficiency. The observed biases were considered undisruptive to the focus of the study to produce a taxonomically broad picture of the BoB planktonic and biofilm communities.

---

[1] In the current study, the chosen primers showed 44% coverage of fungal organisms and ~2% coverage of Ochrophyta organisms, as determined by SILVA TestPrime v1.0 analysis against SILVA SSU v132 database. Similar findings were reported by Prada *et al.* in 2016

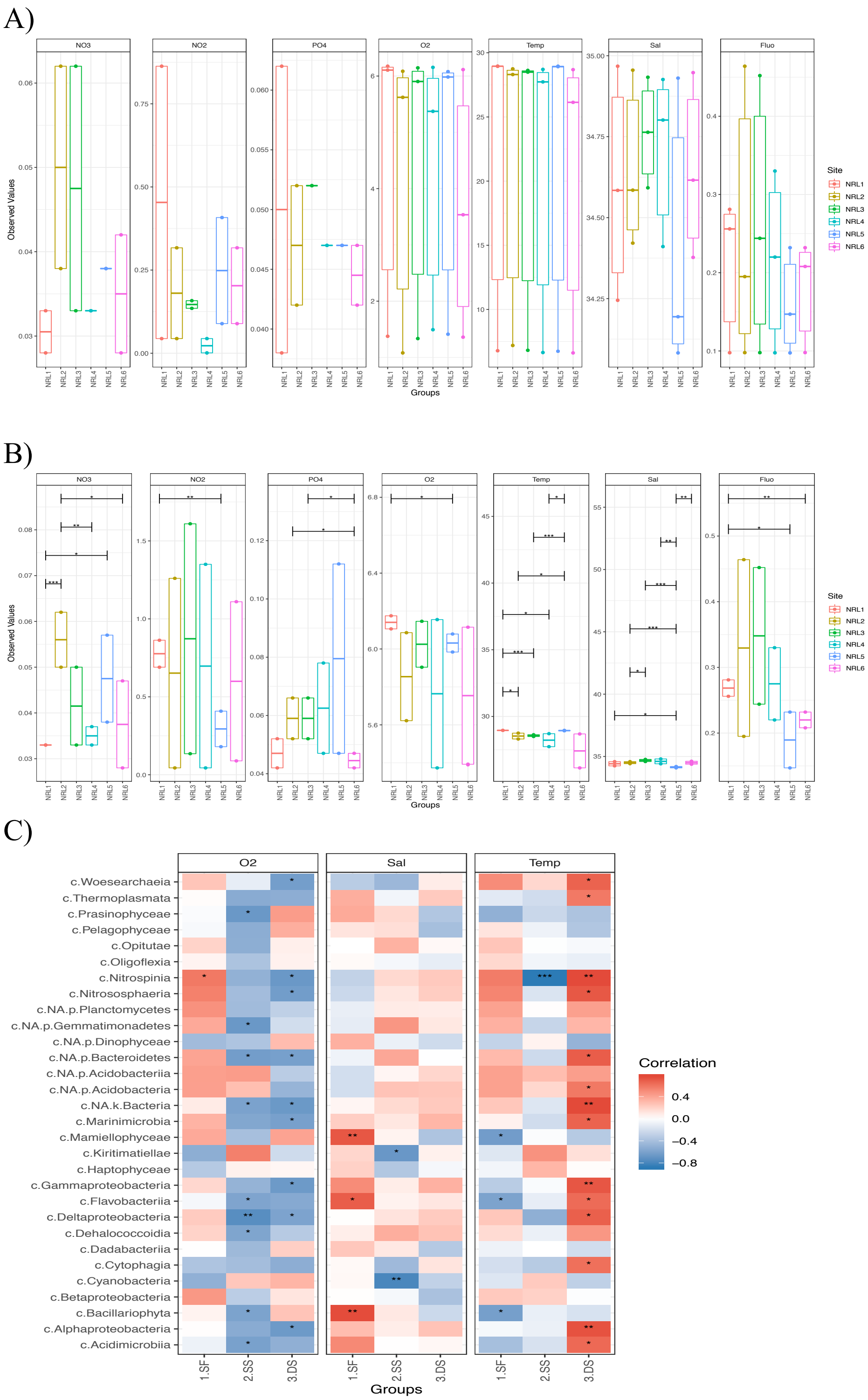

Figure S2. Environmental variables within the BOB water column. A) Observations for changes in variables throughout the entire water column for each site (deep-sea layer measurements for NO2, NO3 and PO4 were unavailable). B) Observations for each site within the photic zone only. C) Taxonomic response to oxygen, salinity and temperature variations throughout the water column. Group abbreviations are as follows: 1.SF – surface water layer, 2.SS – subsurface water layer, 3.DS – deep-sea water layer.

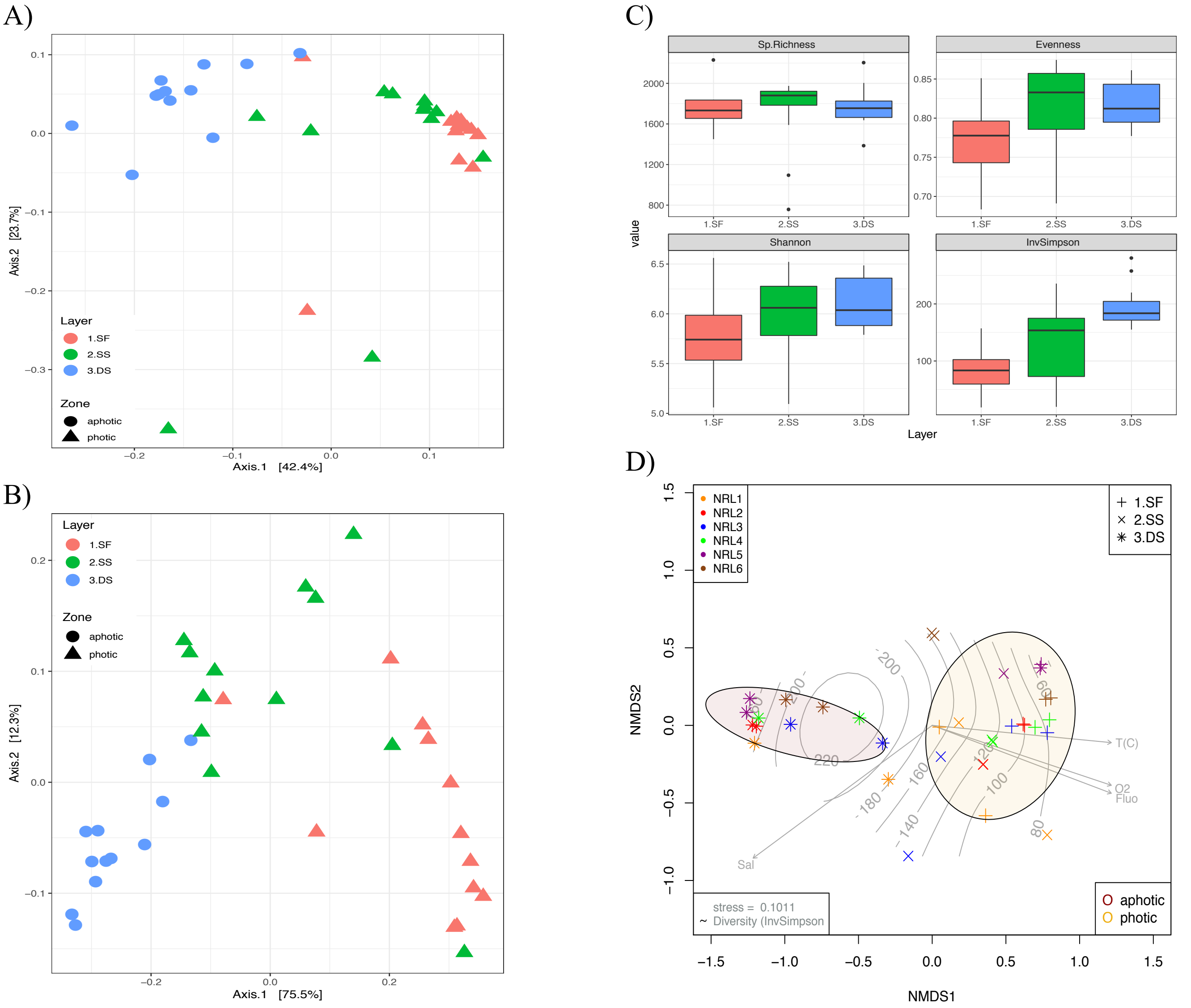

Figure S3. Alpha and beta diversity variances for planktonic microbial communities from BOB. A) Principal Coordinate Analysis (PCoA) based on Unweighted UniFrac (Qualitative) and B) Weighted UniFrac (Quantitative) distances. Dispersion patterns within the plot demonstrate the influence of OTU abundance on the variability of communities. Looser clustering of photic zone samples within the weighted plot show the influence of the most abundant phylotypes within the surface and subsurface layers. The distribution of samples within the deep-sea clusters, demonstrate the influence of the rarer phylotypes within the aphotic zone. Figures were produced based on 16S and 18S rRNA gene fragment sequences produced using universal primers. Numbers in parenthesis on the axis labels designate the percent contribution of each axis to the differences between communities. C) Alpha diversity indices shown for species richness, evenness, Shannon and inverse Simpson, also showing community diversity increasing with depth. D) nMDS ordination using square root normalized Bray-Curtis community matrix values, showing distinguishability between the planktonic BOB communities in the different water layers.

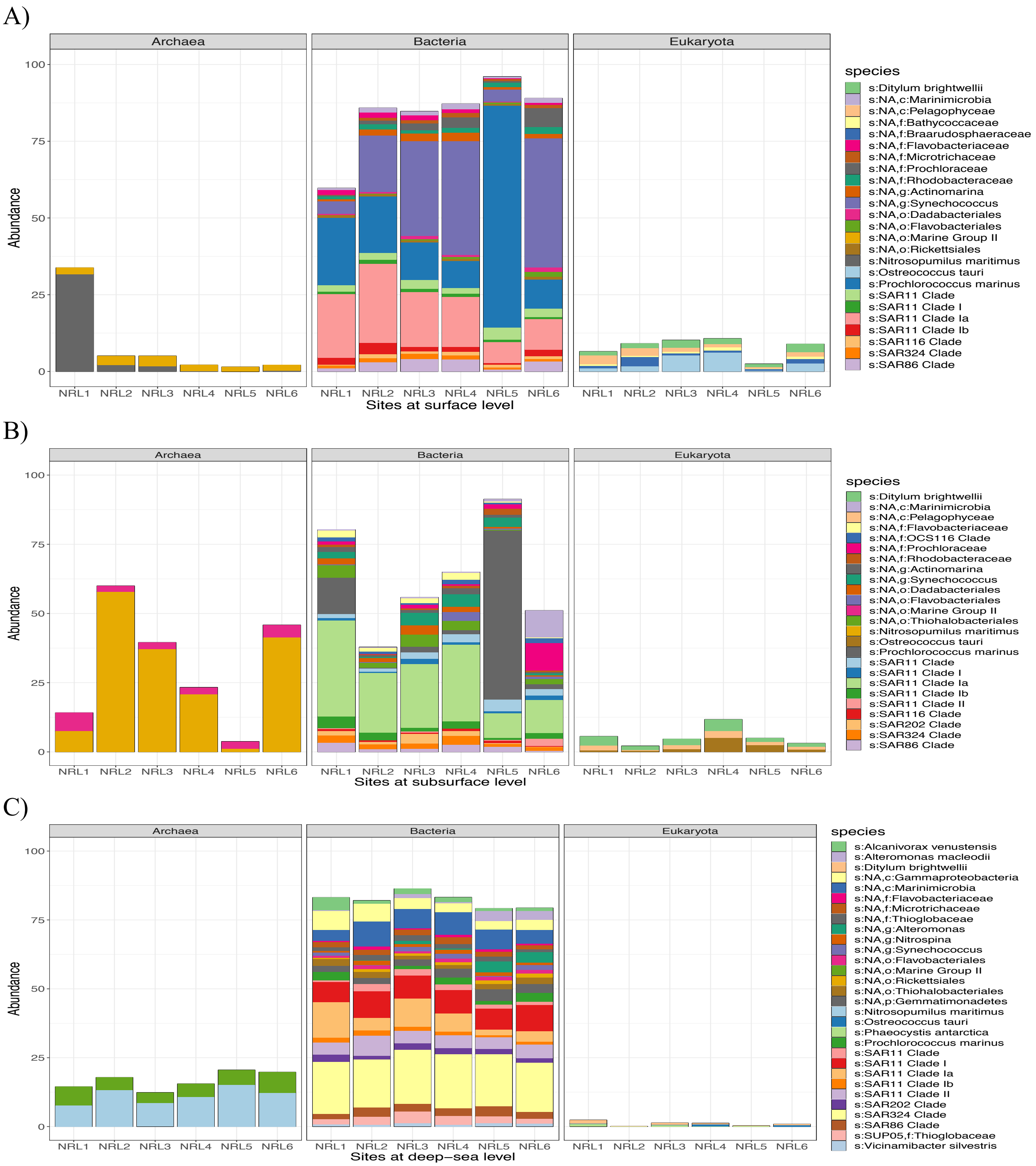

Figure S4. Microbial composition of 25 most abundant species based on 16S rRNA gene amplicons for Bay of Bengal sites NRL1 through NRL6. Profiles were produced based on the average values per 2 biological replicate samples per site for all 6 sites (n=12). The profiles show a *P. marinus* bloom in the surface (A) and photic subsurface (B) layers of the site NRL5, as well as the consistency of deep-sea community composition (C) during the sampled season. Since not all organisms could be classified to species level, some groups remain represented at higher phylogenic ranks.

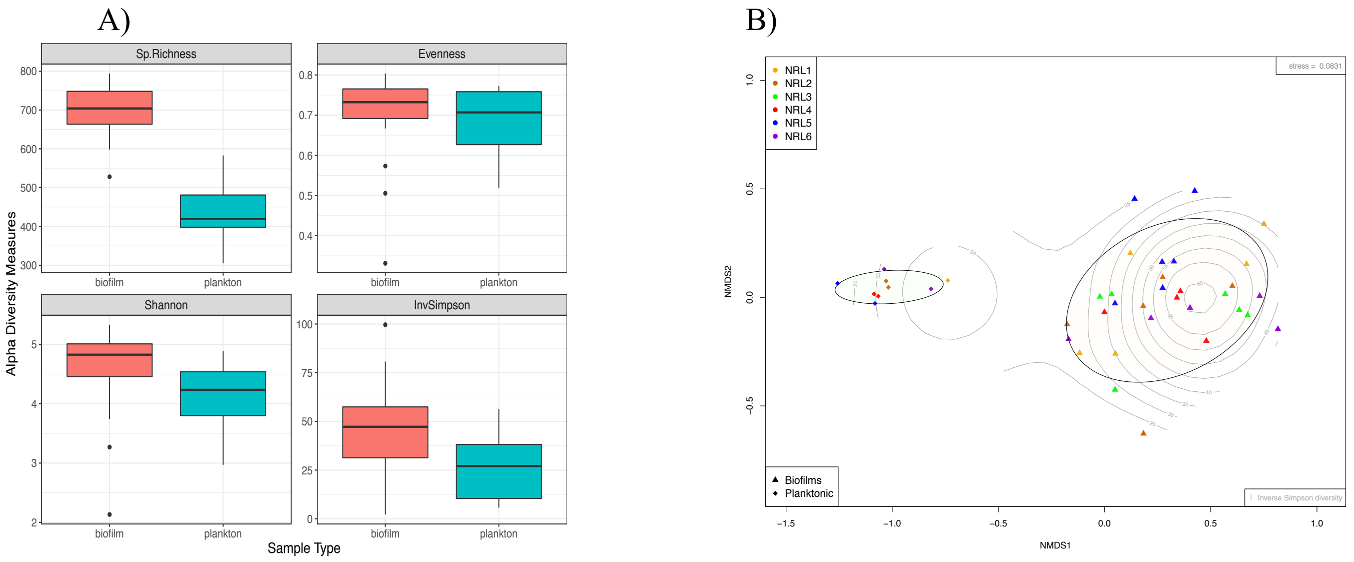

Figure S5. Alpha and Beta diversity analysis plots of the planktonic and co-located biofilm communities at photic subsurface depths (~75m) in Bay of Bengal. A) Alpha diversity indices shown for planktonic *vs*. biofilm communities, including species richness (Sp.Richness), evenness, Shannon and inverted Simpson (Inv.Simpson) (n=24). B) Non-metric multi-dimensional scaling (nMDS) plot of square root transformed Bray-Curtis dissimilarity matrix produced from subsurface planktonic (n = 24) and subsurface biofilm (n = 31) communities. Contoured lines represent Shannon diversity within the water column. Ellipses represent clusters of planktonic and biofilm communities with confidence value of 75%. PERMANOVA analysis p-value = 0.001 for planktonic vs biofilm communities. ANOSIM R – value = 0.89 and p-value = 0.001.

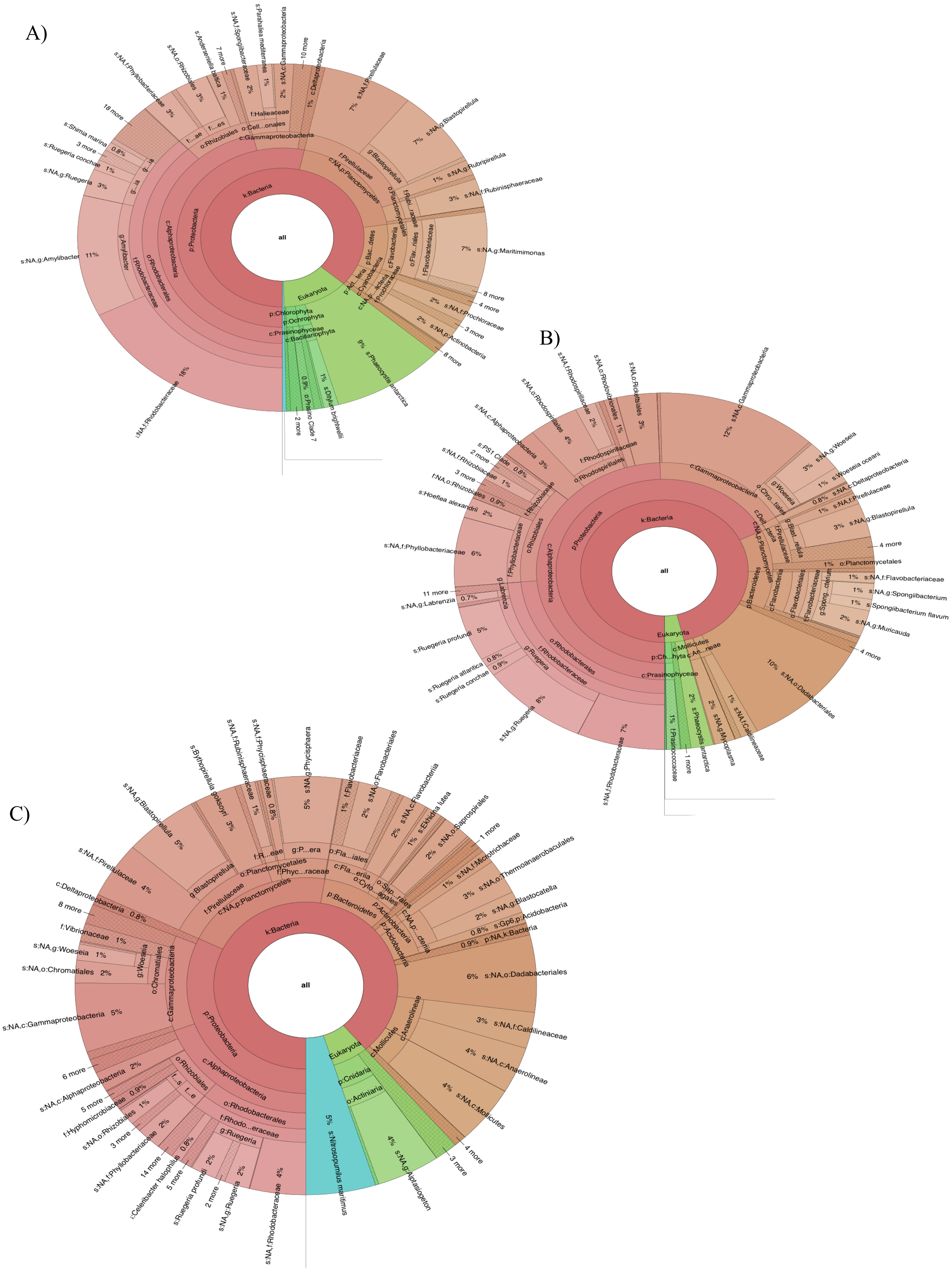

A)
all
k:Bacteria
p:Proteobacteria
c:Alphaproteobacteria
o:Rhodobacterales
f:Rhodobacteraceae
g:Amylibacter
s:NA,g:Amylibacter 11%
s:NA,f:Rhodobacteraceae 18%
c:Gammaproteobacteria
f:Pirellulaceae
c:NA,p:Planctomycetes
g:Blastopirellula
s:NA,g:Blastopirellula 7%
s:NA,f:Pirellulaceae 7%
s:NA,g:Maritimimonas 7%
Eukaryota
p:Chlorophyta
p:Ochrophyta
c:Prasinophyceae
c:Bacillariophyta
s:Phaeocystis antarctica 9%
B)
all
k:Bacteria
p:Proteobacteria
c:Alphaproteobacteria
o:Rhodobacterales
f:Rhodobacteraceae
g:Ruegeria
s:NA,g:Ruegeria 8%
s:NA,f:Rhodobacteraceae 7%
c:Gammaproteobacteria
s:NA,c:Gammaproteobacteria 12%
s:NA,o:Dadabacteriales 10%
s:NA,f:Phyllobacteriaceae 6%
s:Ruegeria profundi 5%
Eukaryota
c:Prasinophyceae
C)
all
k:Bacteria
p:Proteobacteria
c:Alphaproteobacteria
o:Rhodobacterales
c:Gammaproteobacteria
s:NA,c:Gammaproteobacteria 5%
s:NA,g:Blastopirellula 5%
s:NA,g:Phycisphaera 5%
s:NA,o:Dadabacteriales 6%
s:NA,f:Caldilineaceae 3%
s:NA,c:Anaerolineae 4%
s:NA,c:Mollicutes 4%
Eukaryota
p:Cnidaria
o:Actiniaria
s:NA,g:Aiptasiogeton 4%
s:Nitrosopumilus maritimus 5%
s:NA,f:Rhodobacteraceae 4%

D)

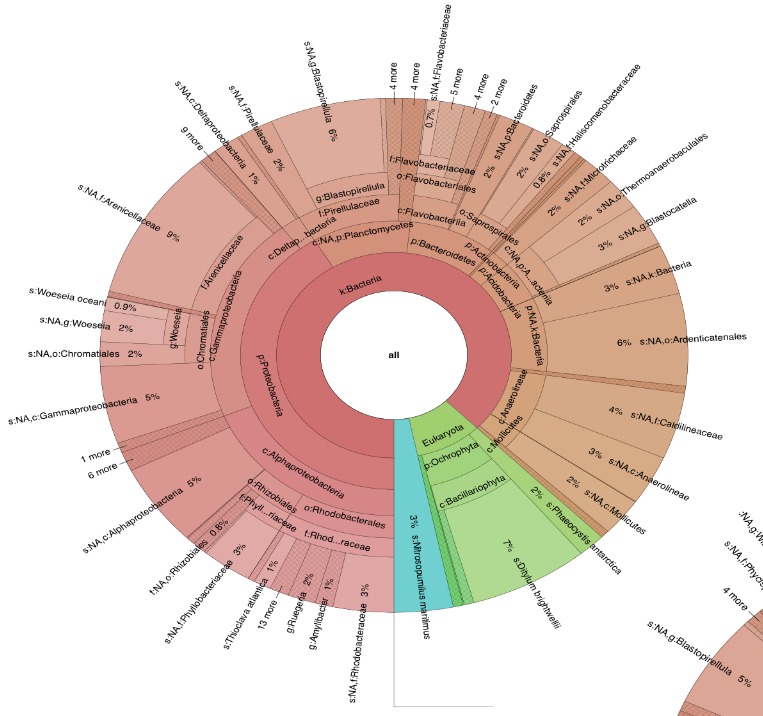

E)

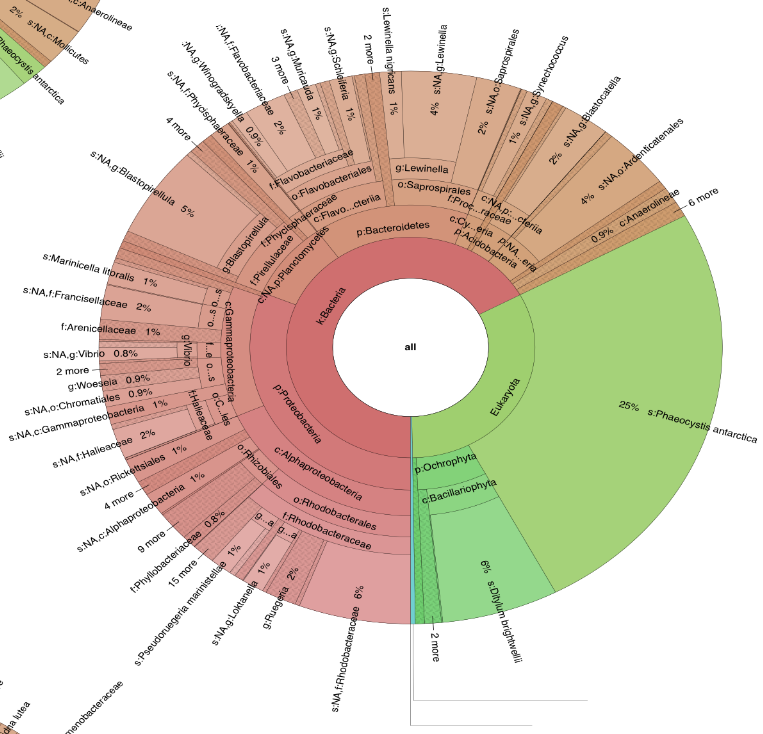

F)

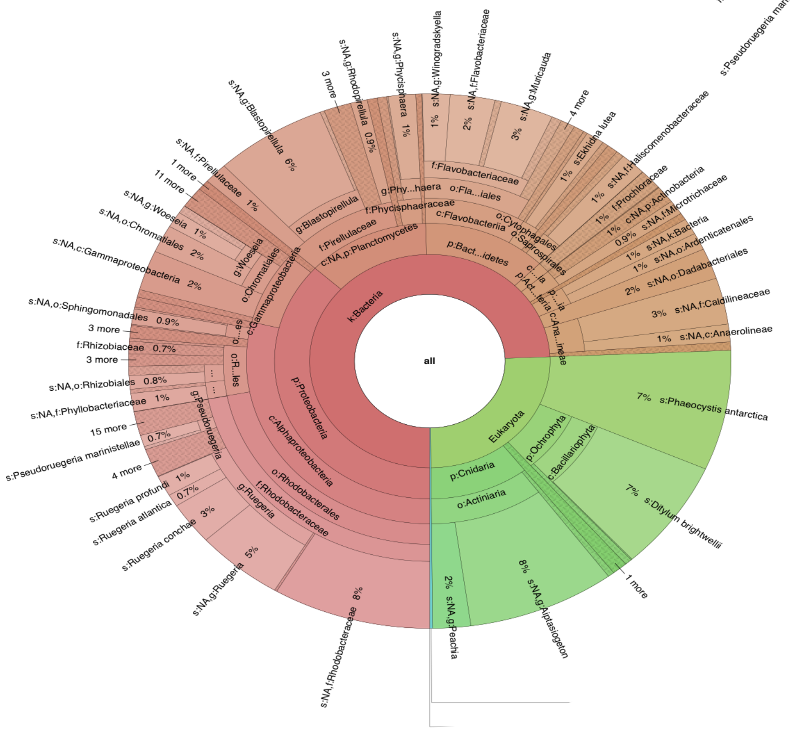

Figure S6. Krona profiles of the 6 clusters of biofilms, down to species level. Profiles producesd by sequencing 16S and 18S rRNA gene fragments with universal primers. Profile A) Cluster 1, B) Cluster 2, C) Cluster 3, D) Cluster 4, E) Cluster 5, F) Cluster 6.

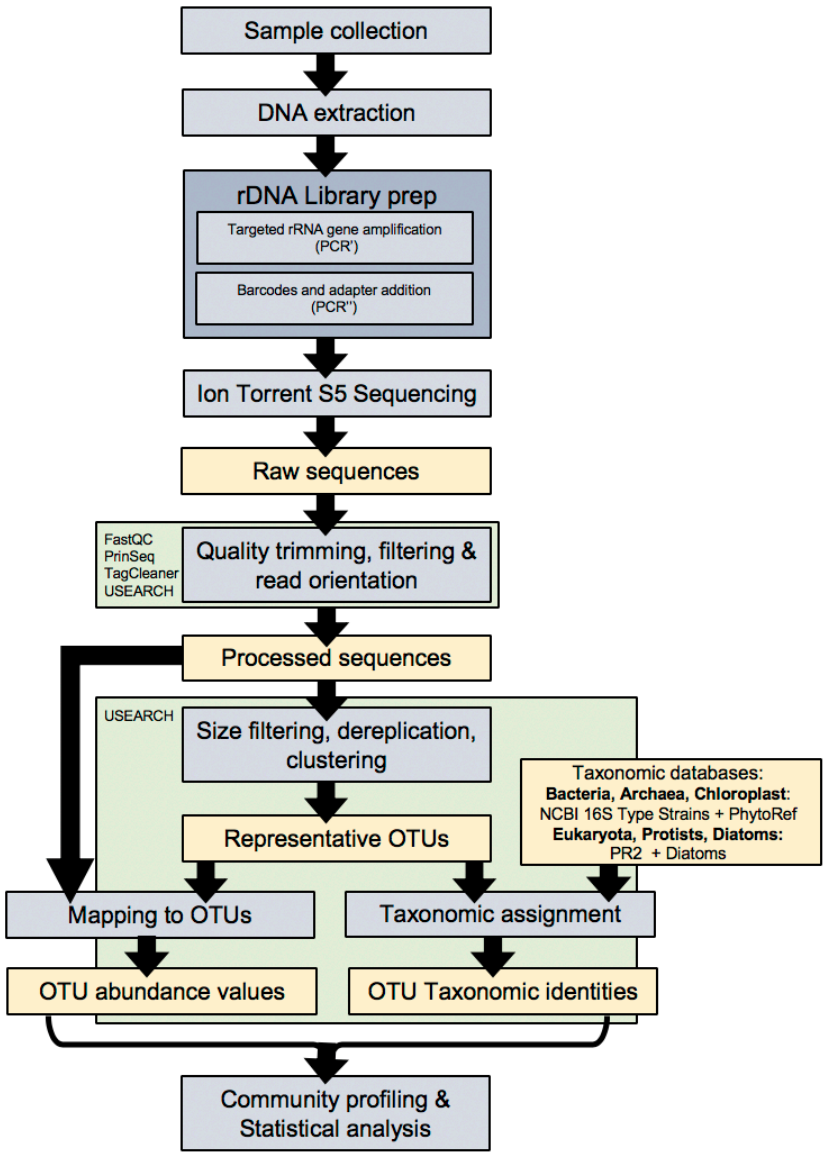

Figure S7: A schematic of the metrological workflow for the BOB community sample exploration. Blue boxes present process names, yellow boxes present data, green boxes summarize tools used for the described steps.
