# Supplementary material for: Microbial Composition and Variability of Natural Marine Planktonic and Biofouling Communities From the Bay of Bengal
Source: Front Microbiol. 2019 Dec 6;10:2738. doi: 10.3389/fmicb.2019.02738 (PMC6908470; doi:10.3389/fmicb.2019.02738)
Supplement: Supplementary file 2 [file Data_Sheet_2.PDF]

Supplementary Table

Table S1. Sequencing and read processing results. Out of 5,075 resulting OTUs, 434 were eukaryotic, 4,376 were bacterial and 264 were archaeal.

Sample Type	SampleID	Sequenced reads	After pre-processing	After OUT mapping	After counts filtering
planktonic samples	NRL1_1000m1	77,816	71,003	40,696	35,434
	NRL1_1000m2	503,588	471,257	323,306	294,656
	NRL1_5m1	1,127,224	1,040,898	648,339	597,741
	NRL1_5m2	588,057	520,818	348,712	309,900
	NRL1_75m1	315,512	287,752	190,755	177,275
	NRL1_75m2	8,512	6,637	3,467	3,453
	NRL2_1000m1	502,677	471,417	329,247	321,850
	NRL2_1000m2	996,082	939,197	672,149	659,946
	NRL2_5m1	850,990	803,736	638,469	636,805
	NRL2_5m2	890,853	841,277	659,386	657,544
	NRL2_75m1	848,732	798,774	639,205	634,566
	NRL2_75m2	898,081	846,982	679,997	675,700
	NRL3_1000m1	197,838	173,926	92,920	86,283
	NRL3_1000m2	258,731	242,673	161,101	154,528
	NRL3_5m1	89,792	81,643	423,766	422,404
	NRL3_5m2	636,738	595,834	477,649	473,603
	NRL3_75m1	96,260	88,825	51,968	41,585
	NRL3_75m2	581,456	545,207	36,866	29,440
	NRL4_1000m1	400,418	373,336	260,665	256,763
	NRL4_1000m2	364,318	338,465	224,932	219,214
	NRL4_5m1	545,194	512,151	416,095	415,052
	NRL4_5m2	708,965	664,052	520,071	518,889
	NRL4_75m1	687,139	640,422	485,298	482,394
	NRL4_75m2	599,602	559,228	426,470	424,190
	NRL5_1000m1	333,605	303,740	176,169	174,106
	NRL5_1000m2	118,204	108,631	63,260	61,815
	NRL5_5m1	683,764	620,293	451,668	451,066
	NRL5_5m2	301,304	267,861	193,117	192,790
	NRL5_75m1	508,867	460,292	320,092	318,605
	NRL5_75m2	382,615	348,283	248,675	248,271
	NRL6_1000m1	156,065	138,616	76,683	74,488
	NRL6_1000m2	265,466	242,290	143,555	137,926
	NRL6_5m1	381,105	353,253	259,251	258,659
	NRL6_5m2	320,387	296,507	215,046	214,613
	NRL6_75m1	375,239	344,185	220,306	213,170
	NRL6_75m2	424,628	389,735	241,296	234,616
biofilm samples	NRL1a	773,029	716,836	502,753	336,018
	NRL1b	126,927	117,573	86,059	66,711
	NRL1c	358,280	334,548	233,029	130,850
	NRL1d	254	235	152	83
	NRL1f	493,129	462,736	328,838	210,340
	NRL1g	486,209	454,469	321,431	229,589
	NRL2a	298,210	277,632	187,052	145,207
	NRL2b	83,572	77,393	58,442	50,934
	NRL2d	189,311	174,331	120,014	98,976
	NRL2e	600	551	411	206
	NRL2f	80,512	75,027	50,742	42,431
	NRL2g	519,541	458,182	331,709	209,998
	NRL3a	1,324,424	1,253,097	1,032,125	398,742
	NRL3d	207,415	189,264	139,673	97,227
	NRL3e	615,232	585,150	489,306	366,232
	NRL3f	633,280	604,067	500,136	319,173
	NRL3g	1,256,063	1,193,173	928,892	380,318
	NRL3h	600,185	564,053	441,464	234,754
	NRL4a	17,766	14,289	9,135	6,505
	NRL4b	293,372	277,007	216,097	150,265
	NRL4c	172,605	160,299	116,983	60,949
	NRL4d	15,692	11,871	8,062	4,649
	NRL4f	189,544	175,037	124,953	56,630
	NRL4g	762,766	714,712	517,539	325,620
	NRL5a	452,920	429,604	331,695	170,375
	NRL5d	731,460	687,311	501,696	313,139
	NRL5e	1,178,471	1,113,675	844,377	356,221
	NRL5f	397,903	379,341	301,383	84,019
	NRL5g	730,100	689,512	480,766	232,514
	NRL5h	335,143	319,334	232,312	136,070
	NRL6a	577,575	548,457	435,085	377,544
	NRL6b	179,316	170,843	129,738	88,605
	NRL6c	24,289	22,064	14,337	9,852
	NRL6f	439,936	416,082	308,397	226,372
	NRL6g	484,259	457,510	330,334	216,769
	NRL6h	621,661	572,058	359,825	236,731
